# Supplementary material for: An Environmental Niche Model to Estimate the Potential Presence of Venezuelan Equine Encephalitis Virus in Costa Rica
Source: Int J Environ Res Public Health. 2020 Dec 30;18(1):227. doi: 10.3390/ijerph18010227 (PMC7795298; doi:10.3390/ijerph18010227)
Supplement: Supplementary file 1 [file ijerph-18-00227-s001.pdf]

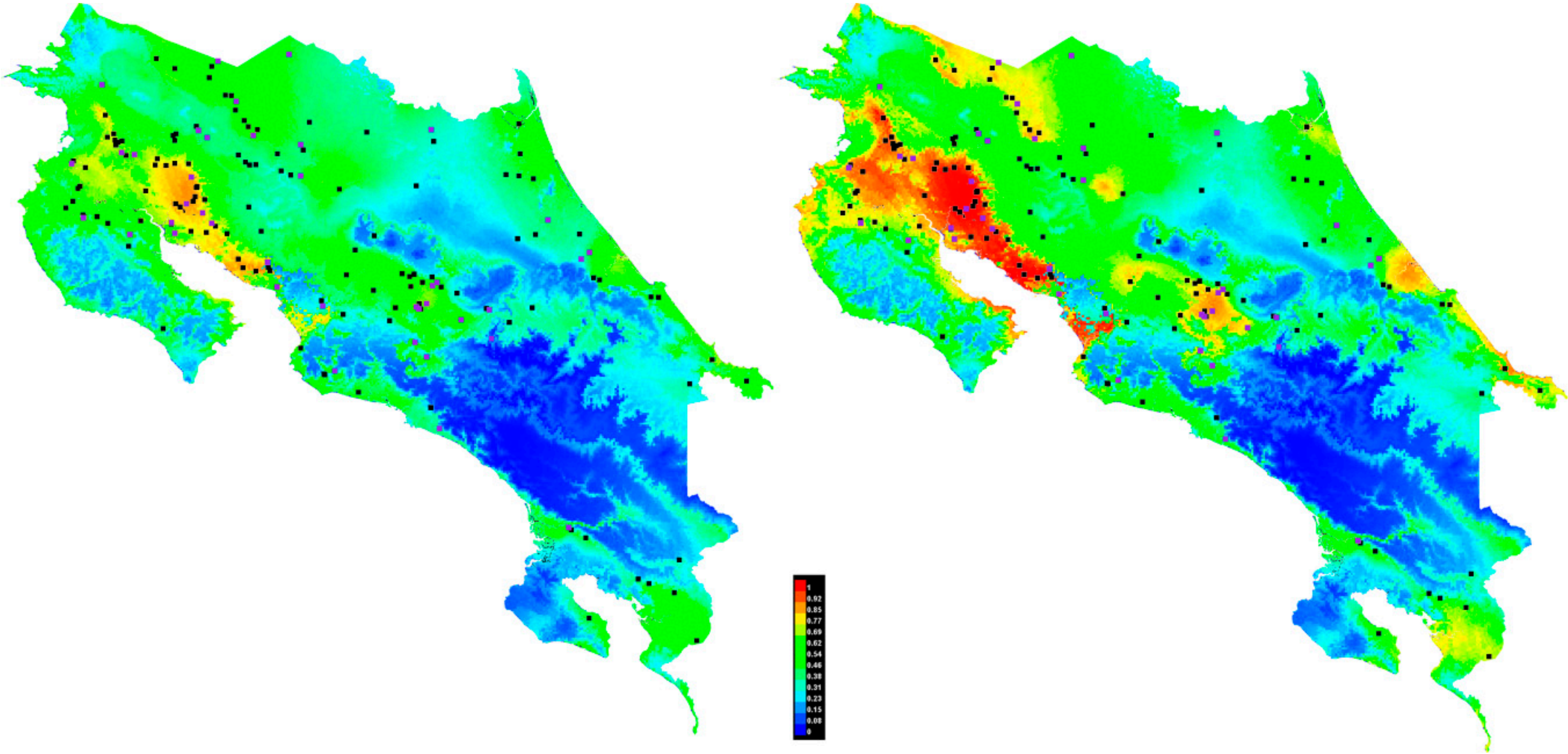

Figure S1. Output map styles produced by MaxEnt, in the left side is presented the logistic output format and in the right is depicted the cloglog output format.
